# High Fat Diet‐Induced Obesity Alters Cutaneous Immune Cell Function, and These Changes Persist After Weight Loss

**DOI:** 10.1155/jimr/3930910

**Published:** 2026-05-15

**Authors:** Wakana Kamada, Hiromasa Tanno, Rena Takayashiki, Yuki Sato, Shinyo Ishi, Miki Shoji, Ko Sato, Tetsuji Aoyagi, Emi Kanno

**Affiliations:** ^1^ Department of Translational Science for Nursing, Tohoku University Graduate School of Medicine, 2-1 Seiryo-machi, Aoba-ku, Sendai, Miyagi, Japan, tohoku.ac.jp; ^2^ Department of Plastic and Reconstructive Surgery, Tohoku University Graduate School of Medicine, 2-1 Seiryo-machi, Aoba-ku, Sendai, Miyagi, Japan, tohoku.ac.jp; ^3^ Department of Clinical Microbiology and Infection, Tohoku University Graduate School of Medicine, 2-1 Seiryo-machi, Aoba-ku, Sendai, Miyagi, Japan, tohoku.ac.jp

**Keywords:** obese, obesity-associated immune memory, skin immunity, weight loss

## Abstract

Obesity is recognized as a chronic low‐grade inflammation that contributes to metabolic disorders. Weight loss (WL) is well known to improve metabolic disorders. However, recent studies have shown that the changes in immune cells associated with obesity exhibit immunological memory even after WL. Obesity impairs skin barrier function and exacerbates inflammatory skin diseases, such as psoriasis. While skin immune cells maintain homeostasis and defense, the impact of obesity on these cells in intact skin is understudied, as research mainly focuses on skin with an inflammatory skin disease model in the context of obesity. The effect of WL on skin immunity is even less clear. Therefore, this study aimed to investigate these discrepancies. Mice were assigned to the lean group (regular diet), the obese group (high‐fat diet for 18 weeks), and the WL group (high‐fat diet for 9 weeks followed by regular diet for 9 weeks). Following 18 weeks of feeding, mouse skin was excised, and immune cell populations within the skin were analyzed using real‐time PCR and flow cytometry. In addition, the effects of WL on psoriasis were examined using an imiquimod‐induced psoriasis mouse model. We observed increased expression of RORγt, IL‐17A, and CCL20 in intact skin in both the obese and WL groups. In the psoriasis model, disease severity was further exacerbated by WL. Moreover, whereas the Vγ4^+^Vγ5^−^ γδ T cell population was increased in the lean group, the Vγ4^−^Vγ5^−^ γδ T cell population remained elevated following WL, similar to levels observed in the obese group. These observations indicated that obesity may imprint a memorized immune response in the skin, which is not abrogated by WL. This study is the first to demonstrate this persistent effect on skin immunity. These findings imply that individuals who have experienced obesity may remain at increased risk for inflammatory skin conditions, even after WL.

## 1. Introduction

Obesity, a significant global health issue, is recognized as a chronic low‐grade inflammation that contributes to metabolic disorders [1]. As the population with obesity increases, more than half of individuals with obesity have attempted to lose weight [2]. Weight loss (WL) is well known to improve metabolic disorders and reduce the risk of obesity‐related diseases [3]. Conversely, recent studies have shown that, even after WL, the changes in immune cells associated with obesity exhibit immunological memory, and systemic inflammation is sustained [4, 5].

Obesity impairs the skin barrier function, which is associated with reduced collagen content [6, 7]. Furthermore, obesity exacerbates inflammatory skin diseases such as atopic dermatitis and psoriasis and is also associated with delayed skin wound healing [8–11]. Various skin immune cells exist in the steady state, playing crucial roles in host defense and the maintenance of tissue homeostasis. Despite the possibility that changes in skin immune cells capable of exacerbating skin diseases may already be present in pre‐disease intact skin, research on the effects of obesity on skin immune cells has primarily focused on atopic dermatitis and psoriasis models. Consequently, the impact of obesity on immune cells in intact skin is limited, and the impact of WL is even less understood.

Specifically, previous reports indicate that obesity alters immune cells in the skin, resulting in increased numbers of T cells and dermal γδ T cells. These changes are associated with the exacerbation of inflammatory skin diseases, such as the Th17‐related disease psoriasis [12]. Also, the involvement of WL on immune cells in the skin resulted in an increase in the number of M1 macrophages and a decrease in the number of M2 macrophages [13]. As indicated above, despite the presence of various cells in the skin, research on the effects of obesity and WL on immune cells in the skin has been limited to a few cell types. Therefore, this study aimed to investigate the effects of obesity and WL on cutaneous immune cells. Furthermore, because the influence of immune cells following WL on inflammatory skin diseases remains unclear, we examined these effects using a psoriasis animal model.

## 2. Materials and Methods

### 2.1. Animals and Mouse Models

**Figure 1:**
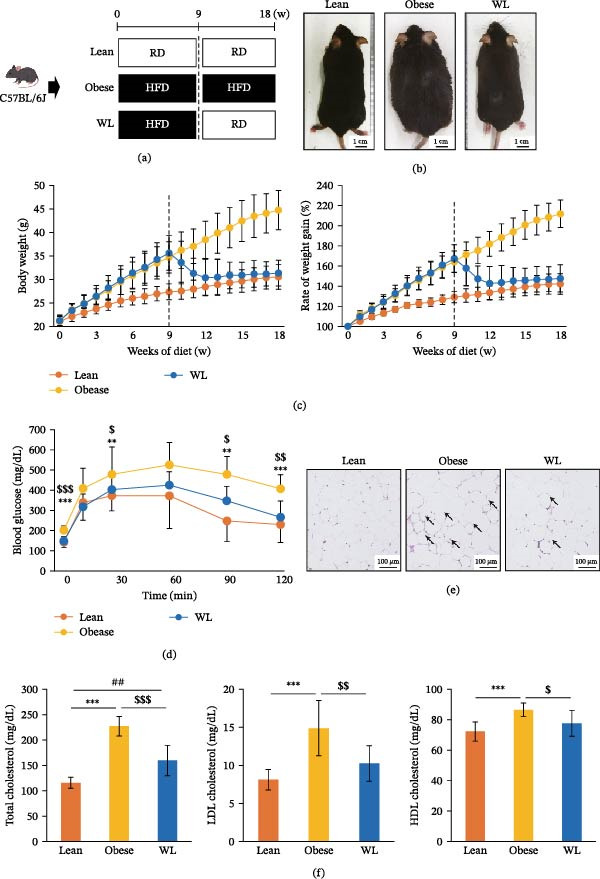
Establishment of the lean, obese, weight loss (WL) groups. (a) The schedules for generating mouse models. (b) Representative photographs of mice from each group after 18 weeks of feeding. (c) Body weight and rate of weight gain. The vertical dotted lines indicate the timing of diet switching (*n* = 24 mice per group). (d) Intraperitoneal glucose tolerance test (ipGTT) at 18 weeks post‐diet initiation (*n* = 12 mice per group). (e) Representative histological images of eWAT. Arrows indicate crown‐like structures. (f) Serum levels of total cholesterol, LDL cholesterol, and HDL cholesterol (*n* = 8 mice per group). Results are representative of at least two independent experiments.  ^∗∗^ *p* < 0.01,  ^∗∗∗^ *p* < 0.001, Lean vs. Obese; ##*p* < 0.01, Lean vs. WL; $ *p* < 0.05, $$ *p* < 0.01, $$$ *p* < 0.001 Obese vs. WL.

Male C57BL/6J mice at 7–8 weeks of age were purchased from CLEA Japan (Tokyo, Japan). Mice were divided into three groups based on their diet: the lean, obese, and WL groups. In accordance with a previous study, the lean group was fed a regular diet (RD; AIN‐93 M; 9% energy from fat; Oriental Yeast Co., Ltd., Tokyo, Japan) for 18 weeks, the obese group was fed a high‐fat diet (HFD; HFD60; 62.2% energy from fat; Oriental Yeast Co., Ltd.) for 18 weeks, and the WL group was fed HFD for 9 weeks and then switched to RD for 9 weeks (total 18 weeks; Figure 1a). Body weights were measured weekly. Mice were housed under specific pathogen‐free conditions at the Institute for Animal Experimentation, Tohoku University Graduate School of Medicine (Sendai, Japan) with *ad libitum* access to food and water. All experimental protocols described in this study were approved by the Ethics Review Committee for Animal Experimentation of Tohoku University (Approval No. 2023‐MdA‐058‐02, 2025‐MdA‐104‐01). All experiments were performed under anesthesia, and every effort was made to minimize the suffering of animals.

### 2.2. Tissue Collection

At the end of the experimental periods, mice were euthanized by isoflurane (Mairan Pharma, Osaka, Japan) inhalation at a lethal concentration (exceeding 5.0%), and respiratory arrest was confirmed before tissue collection. The dorsal skin and the epididymal white adipose tissue (eWAT) were then collected using scissors and a surgical knife.

### 2.3. Histopathology

The dorsal skin and the eWAT were fixed in 4% paraformaldehyde‐phosphate buffer solution and embedded in paraffin after caudocranial dissection, as previously described [14]. Sections were stained with hematoxylin and eosin (H&E) according to the standard method. The staining sections were observed using a System biological microscope BX 53 (Olympus, Tokyo, Japan) and an All‐in‐one fluorescence microscope BZ‐X800 (KEYENCE, Osaka, Japan).

### 2.4. Glucose Tolerance Test

The glucose tolerance test (GTT) was performed at week 18 of feeding. In accordance with a previous study [4], mice were fasted for 5 h, and baseline (fasting) blood glucose levels were measured. Subsequently, a glucose solution (1.5 g/kg body weight) was administered via intraperitoneal (i.p.) injection. Blood glucose levels were then measured at 15, 30, 60, 90, and 120 min post‐injection. Blood glucose levels were determined using a FreeStyle Freedom Lite glucose meter (NIPRO, Osaka, Japan).

### 2.5. Biochemical Assays of Serum Markers

At 18 weeks of feeding, serum was collected from the mice. Serum levels of total cholesterol, LDL cholesterol, and HDL cholesterol were measured by Oriental Yeast Co., Ltd.

### 2.6. Imiquimod‐Induced Psoriasis Model

Mice were anesthetized with isoflurane (induction at 5.0% and maintenance at 2.0%; Mairan Pharma). In accordance with a previous study [15], a psoriasis mouse model was established by shaving the dorsal hair and topically applying 62.5 mg of 5% imiquimod (IMQ) cream (Beselna Cream; Mochida, Tokyo, Japan) to the dorsal skin daily for six consecutive days. Psoriasis severity was evaluated using the mouse Psoriasis Area and Severity Index (PASI) score, calculated as described previously [16]. The severity of erythema, scaling, and thickness were each assessed on a 5‐point scale: 0 (none), 1 (slight), 2 (moderate), 3 (marked), and 4 (very marked). The total PASI score was defined as the sum of the scores for erythema, scaling, and thickness. Transepidermal water loss (TEWL) was measured on the dorsal skin using a Tewameter mobile TM‐M (Courage + Khazaka Electronic GmbH, Cologne, Germany).

### 2.7. RNA Extraction and Real‐Time Quantitative Polymerase Chain Reaction (RT‐PCR)

Total RNA was extracted from the tissues using ISOGEN (Nippon Gene CO. Ltd., Tokyo, Japan). Complementary deoxyribonucleic acid (cDNA) was synthesized using the PrimeScript 1st‐standard cDNA synthesis kit (TaKaRa Bio Inc., Otsu, Japan) according to the manufacturer’s instructions. Real‐time quantitative PCR was performed using gene‐specific primers and FastStart essential DNA green master mix (Sigma–Aldrich, St. Louis, MO, USA) on a StepOnePlus Real‐Time PCR System (Thermo Fisher Scientific, Inc., Waltham, USA). The results were analyzed using a relative quantification procedure and are presented as expression levels relative to that of 18S rRNA. Primers are described in Table 1.

**Table 1:** PCR primers for analysis.

Gene	Forward (5^′^→3^′^)	Reverse (5^′^→3^′^)
18S rRNA	TTCTGGCCAACGGTCTAGACAAC	CCAGTGGTCTTGGTGTGCTGA
COL1A1	TGTTCAGCTTTGTGGACCTCCG	TACCTCGGGTTTCCACGTCTCA
T‐bet	AGAGACCCAGTTCATTGCAGTGAC	GGACACTCGTATCAACAGATGCGTA
GATA3	TTTCGCAGGAGCAGTATCATGAAG	ATTCAGTGGTTGGAATGCAGACA
RORγt	CACAGAGACACCACCGGACAT	CATGCAGGAGTAGGCCACAT
Foxp3	AGTGCCTGTGTCCTCAATGGTC	AGGGCCAGCATAGGTGCAAG
CD4	CTAGTTCCAGGCCCTCGGTA	GGGTGAGAACAGCAGTGATCAA
CD8	AACCACCACTGTGAAATTCCTGTAG	TGCCTGAGTCGTATCTGTCAAACC
CCL20	ATGGGTACTGCTGGCTCACCTC	ACAAGCTTCATCGGCCATCTG
CCR6	GATGCTGCTCCTGGCCTGTA	GCGTCAGTGTTCTGGAGCGTA
IFN‐γ	GGCACAGTCATTGAAAGCCTAGAA	AGAGATAATCTGGCTCTGCAGGA
IL‐4	GATCCGGATGGTCCCATTCTA	CTCTGCAGCTCCATGAGAACACTA
IL‐1β	CCAGGATGAGGACATGAGCAC	TGTTGTTCATCTCGGAGCCTGTA
IL‐6	CCACTTCACAAGTCGGAGGCTTA	TGCAAGTGCATCATCGTTGTTC
IL‐17A	CTCCAGAAGGCCCTCAGACTAC	GGGTCTTCATTGCGGTGG
IL‐23	CTCAGCCAACTCCTCCAGCCAG	CTGCTCCGTGGGCAAAGACC
IL‐22	TCCGCAGCCATACATCGTC	CTTCCAGGGTGAAGTTGAGCA
TNF‐α	ACTCCAGGCGGTGCCTATGT	GTGAGGGTCTGGGCCATAGAA
TGF‐β	CATTGCTGTCCCGTGCAGA	AGGTAACGCCAGGAATTGTTGCTA
IL‐10	GCCAGAGCCACATGCTCCTA	GATAAGGCTTGGCAACCCAAGTAA
iNOS	ATGGCTCGGGATGTGGCTAC	AAAGACTGCACCGAAGATATCTTCA
CD11c	CATCATTCAAGCAGAGCCAGAAC	GCTACCCGAGCCATCAATCA
CD206	GATAGGCATGTTCCGAAATGTTGA	TATTCCAAAGGCCCGAAGATGA

### 2.8. Preparation of Leukocytes in the Skin

The dorsal skins were excised, minced, and digested in RPMI 1640 medium (Sigma–Aldrich) supplemented with 10 mM HEPES, 10% fetal calf serum (FCS; BioWest, Nuaillé, France), 0.2 mg/mL Liberase TL, 2.5 mg/mL collagenase, 0.1 mg/mL DNase, and 2.0 mg/mL Dispase (Sigma–Aldrich) as previously described [17]. The tissues were incubated for 2 h at 37°C with shaking. After incubation, the tissue fragments and most dead cells were removed by passing the cells through a 70‐μm cell strainer (BD Falcon, Bedford, MA, USA). After centrifugation, the cell pellet was resuspended in 4 mL of 40% Percoll (Pharmacia, Uppsala, Sweden) and layered onto 4 mL of 80% Percoll. After centrifugation at 1800 rpm for 20 min at 20°C, the cells at the interface were collected, washed three times, and counted using a hemocytometer. The absolute number of each immune cell subset was calculated by multiplying the total viable leukocyte count (as determined by hemocytometer) by the percentage of each subset obtained from flow cytometric analysis.

### 2.9. Flow Cytometric Analysis

Cells, obtained as described above, were incubated with an anti‐FCγR Ⅱ/Ⅲ monoclonal antibody (mAb; clone 2.4G2, BD BioScience, Franklin Lakes, NJ, USA) on ice for 15 min. Subsequently, the cells were stained with APC/Cyanine7‐anti‐CD45 mAb (clone 30‐F11, BioLegend, San Diego, CA USA), APC‐aniti‐CD3ε mAb (clone 145‐2C11, BioLegend), PerCP/Cyanine5.5‐aniti‐CD3ε mAb (clone 145‐2C11, BioLegend), FITC‐anti‐T‐cell receptor γδ (TCRγδ) mAb (clone GL3, BioLegend), PE‐mouse‐CD1d Tetramer (α‐GalCer loaded; Medical & Biological Laboratories Co., Ltd. (MBL), Tokyo, Japan), PerCP/Cyanine5.5‐anti‐CD45R/B220 mAb (clone RA3‐6B2, BioLegend), PE‐anti‐Vγ4 mAb (clone UC3‐10A6, BioLegend), APC‐anti‐Vγ5 mAb (clone 536, BioLegend), and LIVE/DEAD Fixable Aqua Stain (Thermo Fisher Scientific). Isotype‐matched irrelevant IgG was used for control staining. For the mouse‐CD1d Tetramer (α‐GalCer loaded), CD1d Tetramer (α‐GalCer unloaded; MBL) was used for the control staining.

CD45^+^TCRγδ^-^CD45R/B220^+^ lymphocytes were identified as B cells; CD45^+^TCRγδ^-^CD3^+^CD1d Tetramer(α‐GalCer loaded)^+^ lymphocytes as iNKT cells; CD45^+^CD3^+^ lymphocytes as T cells. γδ T cells (CD45^+^CD3^+^TCRγδ^+^) were further subclassified into three populations based on the expression of Vγ4 and Vγ5: Vγ4^+^Vγ5^−^ population, Vγ4^−^Vγ5^−^ population, and Vγ4^−^Vγ5^+^ population. The stained cells were analyzed using a BD LSRFortessa Cell Analyzer (BD Bioscience, Franklin Lakes, NJ, USA). The gating strategy is shown in Supporting Information 1: Figure S1.

### 2.10. Statistical Analysis

Data are expressed as the mean ± standard deviation (SD). Data analysis was performed using one‐way analysis of variance (ANOVA) with *post hoc* Tukey–Kramer’s honestly significant difference (HSD) test for more than three experimental groups. A *p*‐value less than 0.05 was considered to indicate statistical significance.

Statistically significant results from the analyses are presented in Supporting Information 2: Table S1.

## 3. Results

### 3.1. Establishment of Mouse Models for the Lean, Obese, and Weight Loss Groups

To generate each mouse model, mice in the lean group were fed a regular diet (RD) for 18 weeks, mice in the obese group were fed a high‐fat diet (HFD) for 18 weeks, and the WL mice were fed HFD for 9 weeks and then switched to RD for 9 weeks, as previously described (Figure 1a) [4]. Representative photographs of mice from each group after 18 weeks of feeding are shown (Figure 1b). At week 9, both the obese and WL groups showed significantly higher body weights compared with the lean group (1.27‐fold higher; 34.7 ± 3.3 g and 1.30‐fold higher; 35.6 ± 3.7 g, respectively, vs. 27.4 ± 1.7 g; both *p* < 0.001). By week 18, the body weight of the obese group remained significantly elevated (1.47‐fold higher; 44.7 ± 4.2 g vs. 30.5 ± 2.6 g, *p* < 0.001). In contrast, the WL group showed a significant reduction in body weight compared with the obese group (*p* < 0.001), reaching a level comparable to the lean group (31.3 ± 2.7 g; *p* > 0.05 vs. lean). The rate of weight gain followed a similar pattern, reflecting these changes in absolute body weight (Figure 1c).

In the obese group, blood glucose levels were significantly higher than those in both the lean and WL groups at several time points, including fasting, 30, 90, and 120 min post‐glucose administration. For instance, levels were significantly higher at fasting (201.5 ± 21.3 mg/dL vs. 142.2 ± 24.4 mg/dL; *p* < 0.001) and 120 min (407.0 ± 69.8 mg/dL vs. 228.7 ± 88.2 mg/dL; *p* < 0.001). In contrast, the WL group showed significantly lower glucose levels compared with the obese group (*p* < 0.001 at fasting and *p* < 0.01 at 120 min), measuring 148.5 ± 20.9 mg/dL at fasting and 265.7 ± 80.9 mg/dL at 120 min, respectively. These values were not significantly different from those of the lean group throughout the test (*p* > 0.05; Figure 1d).

Consistent with a previous study [18], crown‐like structures were observed in the visceral fat of the obese group and, while reduced, persisted in the WL group (Figure 1e). Serum levels of total cholesterol, LDL cholesterol, and HDL cholesterol were significantly increased in the obese group compared with the lean group (all *p* < 0.001). In the WL group, these parameters were significantly reduced compared with the obese group (all *p* < 0.01) and were comparable to lean group levels (*p* > 0.05), with the exception of total cholesterol, which remained significantly higher than in the lean group (*p* < 0.001; Figure 1f).

Based on these results, we concluded that mice in the obese and WL groups were successfully generated and proceeded to analyze the skin tissue.

### 3.2. Persistent Upregulation of Th17‐Associated Genes in the Skin of Mice With a History of Obesity

**Figure 2:**
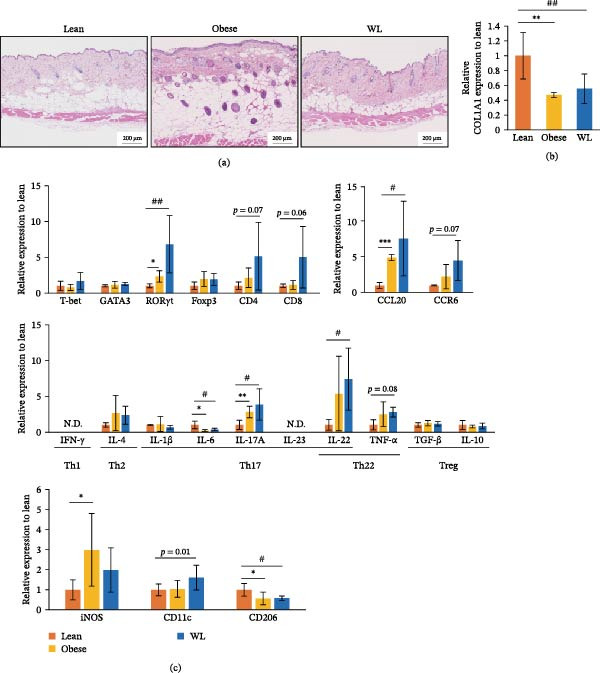
Effects of obesity and weight loss on immune cells in intact skin. (a) Representative histological images of intact skin. (b) The expression of COL1A1 mRNA in intact skin (*n* = 6 mice per group). (c) The expression of T‐bet, GATA3, RORγt, Foxp3, CD4, CD8, IFN‐γ, IL‐4, IL‐1β, IL‐6, IL‐17A, IL‐23, IL‐22, TNF‐α, TGF‐β, IL‐10, CCL20, CCR6, iNOS, CD11c, CD206 mRNA in intact skin (*n* = 6 mice per group). Each column represents the mean ± standard deviation (SD). Results are representative of at least two independent experiments.  ^∗^ *p* < 0.05,  ^∗∗^ *p* < 0.01,  ^∗∗∗^ *p* < 0.001, Lean vs. Obese; # *p* < 0.05, ## *p* < 0.01, Lean vs. WL.

Pathological skin observations showed changes in skin tissue in the obese group, returning to a state comparable with the lean group in the WL group. However, COL1A1, which is a marker for type I collagen, expression was significantly lower in both the obese and WL groups compared with mice in the lean group (0.47 ± 0.033‐fold and 0.55 ± 0.197‐fold, respectively; both *p* < 0.01) (Figure 2a,b). We next examined the expression of genes associated with helper T‐cell subsets (Th1: T‐bet, IFN‐γ; Th2: GATA3, IL‐4; Th17: RORγt, IL‐1β, IL‐6, IL‐17A, IL‐23, IL22; Th22: IL‐22, TNF‐α; Treg: Foxp3, IL‐10, TGF‐β), dermal γδ T cells (CCL20, CCR6), and macrophages (M1: iNOS, CD11c; M2: CD206). There were no significant differences among the groups in the expression of T‐bet, GATA3, and Foxp3. Conversely, the expression of RORγt was significantly increased in the obese group (2.33 ± 0.79‐fold, *p* < 0.05) and the WL group (6.83 ± 3.99‐fold, *p* < 0.01) compared with the lean group. Furthermore, compared with the lean group, IL‐6 expression was significantly decreased in the obese (0.25 ± 0.14‐fold, *p* < 0.05) and WL (0.35 ± 0.17‐fold, *p* < 0.05) groups, IL‐17A expression was significantly increased in the obese (2.81 ± 0.79‐fold, *p* < 0.01) and WL (3.85 ± 2.18‐fold, *p* < 0.05) groups, and IL‐22 expression was significantly increased in the WL group (7.41 ± 4.33‐fold, *p* < 0.05). Additionally, we examined the expression of CCL20 and CCR6, which are implicated in recruiting dermal γδ T cells to the skin. CCL20 expression was significantly increased in both the obese (4.96 ± 0.42‐fold, *p* < 0.001) and WL (7.66 ± 5.29‐fold, *p* < 0.05) groups compared with mice in the lean group. CCR6 expression tended to be higher in the WL group compared with the lean group (4.56 ± 2.83‐fold, *p* = 0.07). In addition, we examined the expression of iNOS, CD11c, and CD206, markers of M1 and M2 macrophages, respectively. iNOS expression was significantly increased in the obese group compared with the lean group (2.99 ± 1.81‐fold, *p* < 0.05). CD206 expression was significantly decreased in both the obese (0.57 ± 0.32‐fold, *p* < 0.05) and WL (0.59 ± 0.11‐fold, *p* < 0.05) groups compared with the lean group (Figure 2c).

### 3.3. Altered Proportions of Dermal γδ T Cells and DETCs in Skin of Weight‐Loss Mice

**Figure 3:**
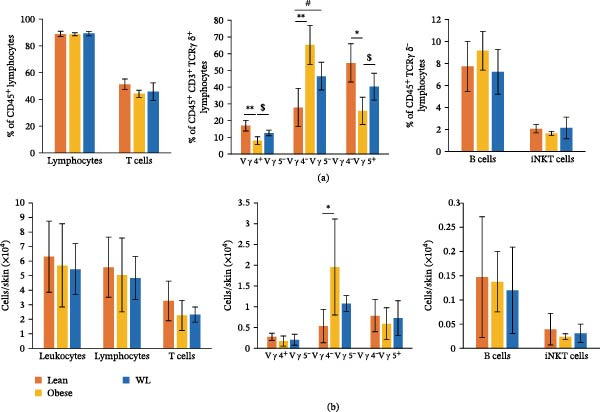
Effects of obesity and weight loss on the lymphocyte fraction in intact skin. (a) The percentage of lymphocytes, T cells, dermal γδ T cells (Vγ4^+^Vγ5^−^, Vγ4^-^Vγ5^−^), DETCs (Vγ4^−^Vγ5^+^), B cells, and iNKT cells in intact skin (*n* = 6 mice per group). (b) The number of leukocytes, lymphocytes, T cells, dermal γδ T cells (Vγ4^+^Vγ5^−^, Vγ4^−^Vγ5^−^), DETCs (Vγ4^−^Vγ5^+^), B cells, and iNKT cells in intact skin (*n* = 6 mice per group). Each column represents the mean ± standard deviation (SD). Results are representative of at least two independent experiments.  ^∗^ *p* < 0.05,  ^∗∗^ *p* < 0.01, Lean vs. Obese; #*p* < 0.05, Lean vs. WL; $ *p* < 0.05, Obese vs. WL.

As shown in Figure 3 and Supporting Information 3: Figure S2, we analyzed the fractions of T cells, B cells, and invariant natural killer T (iNKT) cells in intact skin. γδT cells were further subclassified into three subsets based on the expression of Vγ4 and Vγ5 to specifically identify dermal Vγ4^+^ and Vγ4^−^ γδ T cells, as well as epidermal dendritic T cells (DETCs), which are Vγ5^+^ [19, 20]. The proportions of Vγ4^+^Vγ5^−^ dermal γδ T cells and Vγ4^−^Vγ5^+^ DETCs were significantly decreased in the obese group (Vγ4^+^Vγ5^−^ cells: 0.48‐fold, 8.1 ± 2.2% vs. 16.9 ± 3.3%, *p* < 0.01; Vγ4^−^Vγ5^+^ cells: 0.47‐fold, 25.8 ± 8.1% vs. 54.5 ± 13.4%, *p* < 0.05). These decreases were restored in the WL group (0.75‐fold and 0.74‐fold of the lean group levels, respectively; 12.6 ± 1.7% and 40.3 ± 7.6%), reaching levels that were not significantly different from the lean group (*p* > 0.05). Conversely, the proportion of Vγ4^−^Vγ5^−^ dermal γδ T cells was significantly increased in both the obese (2.34‐fold, 65.3 ± 11.7%) and WL (46.6 ± 8.4%) groups compared with the lean group (1.67‐fold, 27.9 ± 12.5%) (*p* < 0.01 and *p* < 0.05, respectively) (Figure 3a). The proportions of the other analyzed cell populations showed no significant differences among the three groups (Figure 3a).

Furthermore, the absolute number of Vγ4^−^Vγ5^−^ dermal γδ T cells was significantly elevated in the obese group compared with the lean group (3.70‐fold; 1.96 ± 1.16 × 10^4^ cells vs. 0.53 ± 0.40 × 10^4^ cells, *p* < 0.05), whereas the absolute numbers of the other analyzed cell populations did not differ significantly among the three groups (Figure 3b).

### 3.4. Effect of Weight Loss on a Murine Psoriasis Model and Associated γδ T Cell Alterations

**Figure 4:**
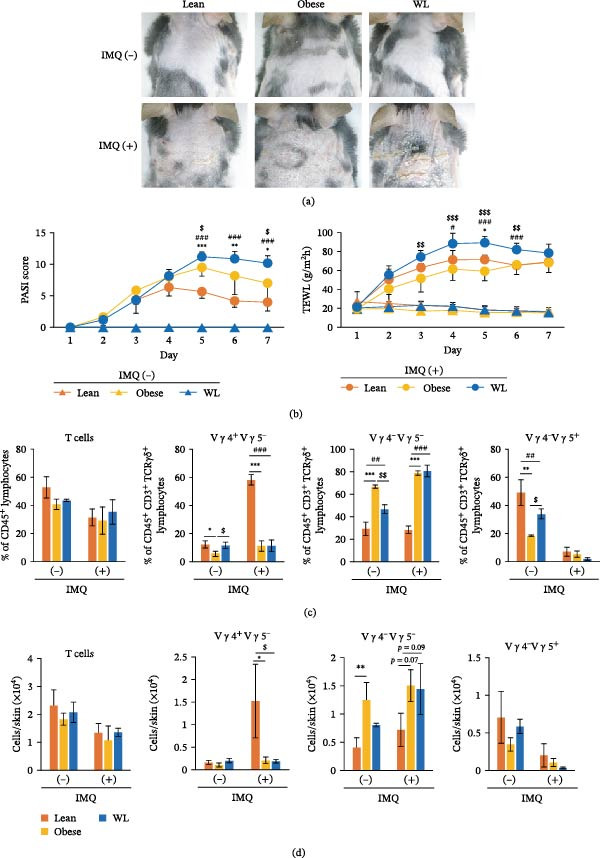
Effect of weight loss on a murine model of psoriasis and alterations in γδ T cells. (a) Representative photograph on day 7 following topical application of 62.5 mg of imiquimod (IMQ). (b) Time course of Psoriasis Area and Severity Index (PASI) scores and transepidermal water loss (TEWL) in skin lesions in the IMQ‐induced psoriasis model. (c) Percentage of T cells, dermal γδ T cells (Vγ4^+^Vγ5^−^, Vγ4^−^Vγ5^−^), and DETCs (Vγ4^−^Vγ5^+^) (*n* = 6 mice per group). (d) Number of T cells, dermal γδ T cells (Vγ4^+^Vγ5^−^, Vγ4^−^Vγ5^−^), and DETCs (Vγ4^−^Vγ5^+^) (*n* = 6 mice per group). Each column represents the mean ± standard deviation (SD). Results are representative of at least two independent experiments.  ^∗^ *p* < 0.05,  ^∗∗^ *p* < 0.01,  ^∗∗∗^ *p* < 0.001, Lean vs. Obese; # *p* < 0.05, ## *p* < 0.01, ### *p* < 0.001 Lean vs. WL; $ *p* < 0.05, $$ *p* < 0.01, Obese vs. WL.

Finally, using a murine model of psoriasis, a Th17‐associated disorder known to be exacerbated by obesity [12], we analyzed whether psoriasis exacerbation persists following WL. PASI scores were significantly higher in both the obese and WL groups than in the lean group on days 5, 6, and 7, with the most pronounced differences observed on day 5 (9.50 ± 1.38 and 11.17 ± 0.75 vs. 5.67 ± 1.03, respectively; both *p* < 0.001 vs. the lean group). Moreover, PASI scores were significantly higher in the WL group than in the obese group on day 5 (*p* < 0.05) and day 7 (10.17 ± 1.17 vs. 7.00 ± 2.53; *p* < 0.05). There were no differences in TEWL, an indicator of skin barrier function, among the three groups prior to IMQ application (Day 1). However, TEWL was significantly increased in the WL group compared with both the lean and obese groups from day 4 through day 6 following IMQ application. Notably, on day 5, when the difference among the groups was most evident, TEWL was significantly higher in the WL group than in the other groups (89.0 ± 6.8 g/m^2^/h vs. 59.2 ± 10.0 g/m^2^/h in the obese group and 71.3 ± 4.3 g/m^2^/h in the lean group; both *p* < 0.001). (Figure 4a,b).

Furthermore, while Vγ4^+^Vγ5^−^ dermal γδ T cells increased in the lean group after IMQ application, the proportion of Vγ4^−^Vγ5^−^ dermal γδ T cells was significantly higher in both the obese and WL groups compared with the lean group (2.77‐fold, 78.8 ± 1.9% and 2.84‐fold, 80.7 ± 5.2% vs. 28.4 ± 3.3%, respectively; both *p* < 0.001) (Figure 4c,d, and Supporting Information 4: Figure S3).

## 4. Discussion

This study utilized a WL model induced by diet switching. In this model, we observed an approximate 10% reduction in body weight within 1 week following the diet switch (week 10). Furthermore, compared with the obese group, which continued on an HFD, the WL group ultimately exhibited an ~20% difference in the rate of weight gain by the final time point (week 18). It has been reported that a 10% reduction in body weight improves metabolic status in individuals with obesity [21, 22]. Consistent with this, our murine model also demonstrated metabolic improvements, including improved glucose tolerance and reduced cholesterol levels. However, despite these metabolic improvements, the proportion of immune cells altered by obesity did not normalize following WL.

Previously, the persistence of obesogenic memory in immune cells within adipose tissue has been reported in both individuals with obesity and murine models [4, 23]. This retained immune memory in adipose macrophages has been shown to have a dual nature, being associated with detrimental effects, such as an increased risk of diabetes following weight regain, as well as potentially protective effects, including a reduced bacterial burden during systemic infection [24]. In contrast, several studies suggest that WL may resolve obesity‐associated immune memory. For example, reductions in plasma B cell activation markers and IgG levels have been reported following bariatric surgery [25]. These findings suggest that the effect of WL on immune memory may be organ‐specific. Indeed, Schmitz et al. [26] reported that obesity‐induced inflammation resolves in the liver but persists in adipose tissue following WL.

Previous studies have reported that obesity decreases skin collagen and that M1 macrophages increase, while M2 macrophages decrease even after WL [7, 13]. In our study, we observed increased expression of RORγt, IL‐17A, and CCL20 in the intact skin of both the obese and WL groups. Furthermore, we observed increased expression of CCR6 and an increase in Vγ4^−^Vγ5^−^ dermal γδ T cells, a potential source of IL‐17A, in the skin of WL group. Our study observed results similar to those of previous models. These observations indicated that obesity may imprint a memorized Th17 response in the skin, which is not abrogated by WL, suggesting that the skin, like adipose tissue, can retain long‐term obesogenic immune memory.

The impact of obesity and WL on immune cells has been comprehensively analyzed in adipose tissue [4]. Cottam et al. [4] reported that obesity leads to T‐cell exhaustion and an increase in inflammatory macrophages, which persist even after WL in adipose tissue. Moreover, Zou et al. [27] reported that CD4^+^ T cells play a role in mediating obesity‐associated immune memory. To the best of our knowledge, this report is the first to demonstrate the persistent effect of obesity on skin immunity even after WL.

As indicated above, obesity is well known to exacerbate inflammatory skin diseases, including atopic dermatitis and psoriasis, and impairs wound healing [8–11]. It is known that the increased Th17 response observed in the obese group in this study was involved in the onset and exacerbation of these diseases. Thus, the potential changes in immune cells in intact skin caused by obesity may contribute to developing and worsening these diseases. Moreover, as the alterations in intact skin immunity caused by obesity persisted following WL, these risks may also continue. In this study, we examined the effects of WL on psoriasis and found that disease severity remained exacerbated following WL, similar to that observed in the obese group. Furthermore, WL failed to normalize obesity‐induced cellular changes in psoriasis, which remained comparable to those in the obese group.

In the lean group, the Vγ4^＋^Vγ5^−^ subset of dermal γδ T cells increased following psoriasis induction. In contrast, in the obese and WL groups, the Vγ4^−^Vγ5^−^ subset of dermal γδ T cells was increased. Dermal γδ T cells are generally classified into Vγ4 and Vγ6 subsets [19]. Although Vγ6 was not directly analyzed by flow cytometry in this study, the Vγ4^−^Vγ5^−^ population is considered to represent Vγ6^+^ γδ T cells. Both Vγ4^+^ and Vγ6^+^ subsets have been reported to contribute to psoriasis pathogenesis [19, 28]. However, it remains unclear which subset is more strongly associated with disease severity. Therefore, further studies are required to determine whether the Vγ4^−^Vγ5^−^ (potentially Vγ6^+^) cells observed in this study play a critical role in the exacerbated psoriatic symptoms in the WL group. In addition, further investigation is necessary to determine whether obesity‐associated immune memory negatively impacts inflammatory skin diseases and wound healing following WL.

This study has several limitations. In this study, we utilized mice in the diet‐induced obese and WL groups. Results may differ when using other models created by different methods, such as exercise‐induced WL mice. Further research is needed to investigate these potential variations.

Furthermore, Hata et al. [5] reported the involvement of epigenetic modifications, such as histone modifications, in the persistence of obesity‐related alterations following WL. Similar epigenetic changes may be involved in the current study, and it will be necessary to conduct further mechanistic studies using scRNA‐seq and ATAC‐seq in the future.

## 5. Conclusion

In conclusion, obesity may induce immunological memory in the skin, specifically a Th17 response, that may not be reversed by WL. This sustained immune alteration may contribute to the continued exacerbation of psoriasis, even after WL. These findings suggest that, even after WL, individuals with a history of obesity may remain at increased risk for inflammatory skin conditions.

## Author Contributions

Funding acquisition: Hiromasa Tanno and Emi Kanno. Project administration: Hiromasa Tanno. Supervision: Hiromasa Tanno, Tetsuji Aoyagi, and Emi Kanno. Formal analysis: Wakana Kamada, Hiromasa Tanno, Rena Takayashiki, Yuki Sato, Shinyo Ishi, Miki Shoji, and Ko Sato. Investigation: Wakana Kamada, Hiromasa Tanno, and Rena Takayashiki. Writing—original draft: Wakana Kamada and Hiromasa Tanno. Writing—review and editing: Wakana Kamada, Hiromasa Tanno, Rena Takayashiki, Yuki Sato, Shinyo Ishi, Miki Shoji, Ko Sato, Tetsuji Aoyagi, and Emi Kanno.

## Acknowledgments

This work was supported in part by a Grant‐in‐Aid for Challenging Research (Exploratory; JP24K22222). A part of this study was supported by the Support System for Young Researchers to use research equipment, instruments, and devices at Tohoku University. During the preparation of this manuscript, Gemini (Google) was used to assist in refining technical terminology to ensure the consistent use of person‐first language. Additionally, the manuscript was reviewed and edited by a professional English editing service to ensure linguistic accuracy.

## Funding

This work was supported by a Grant‐in‐Aid for Challenging Research (Exploratory; JP24K22222).

## Disclosure

All authors reviewed and approved the final manuscript.

## Conflicts of Interest

The authors state no conflicts of interest.

## Supporting Information

Additional supporting information can be found online in the Supporting Information section.

## Supporting information


**Supporting Information 1** Figure S1: Gating strategy of lymphocyte fraction. Gating strategy of flow cytometry for lymphocyte fraction in intact skin.

**Supporting Information 2** Table S1: Detailed results of statistical analyses for all experiments.

**Supporting Information 3** Figure S2: The plots of lymphocyte fraction. The representative plots of T cells, dermal γδ T cells (Vγ4^+^Vγ5^−^, Vγ4^−^Vγ5^−^), DETCs (Vγ4^−^Vγ5^+^), B cells, and iNKT cells in intact skin.

**Supporting Information 4** Figure S3: Representative plots of T cell and γδ T cell fractions on day 7 of IMQ application. Representative plots of T cells, dermal γδ T cells (Vγ4^+^Vγ5^−^, Vγ4^−^Vγ5^−^), and DETCs (Vγ4^−^Vγ5^+^) in both IMQ (‐) and IMQ (+) skin.

## Data Availability

Data from this study are available from the corresponding author upon reasonable request.
