# Supplementary figures and images for: High Fat Diet‐Induced Obesity Alters Cutaneous Immune Cell Function, and These Changes Persist After Weight Loss
Source: J Immunol Res. 2026 May 15;2026:3930910. doi: 10.1155/jimr/3930910 (PMC13177834; doi:10.1155/jimr/3930910)

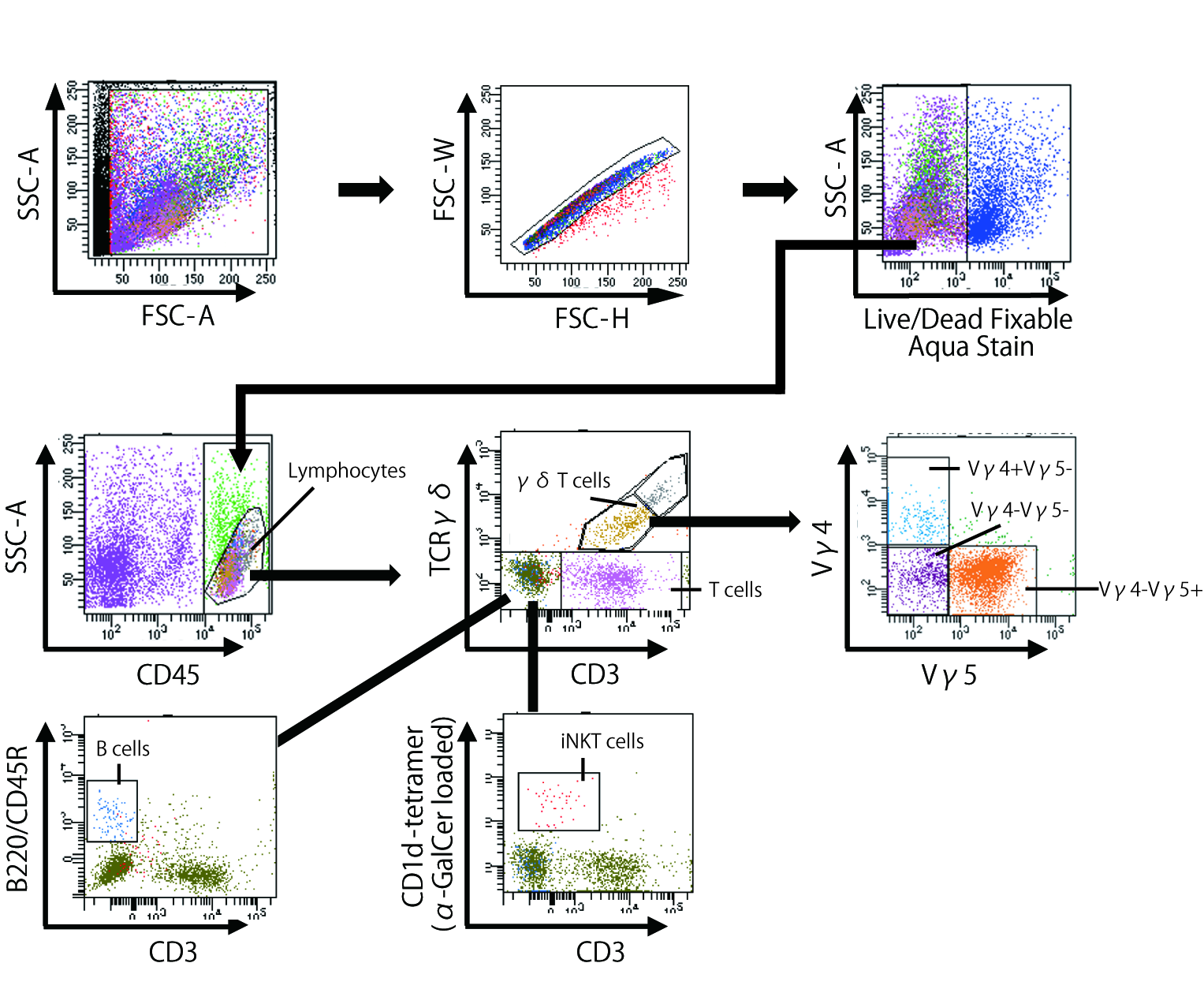

Supplement: Supplementary file 1 — Supporting Information 1 Figure S1: Gating strategy of lymphocyte fraction. Gating strategy of flow cytometry for lymphocyte fraction in intact skin. [file JIMR-2026-3930910-s001.tif]

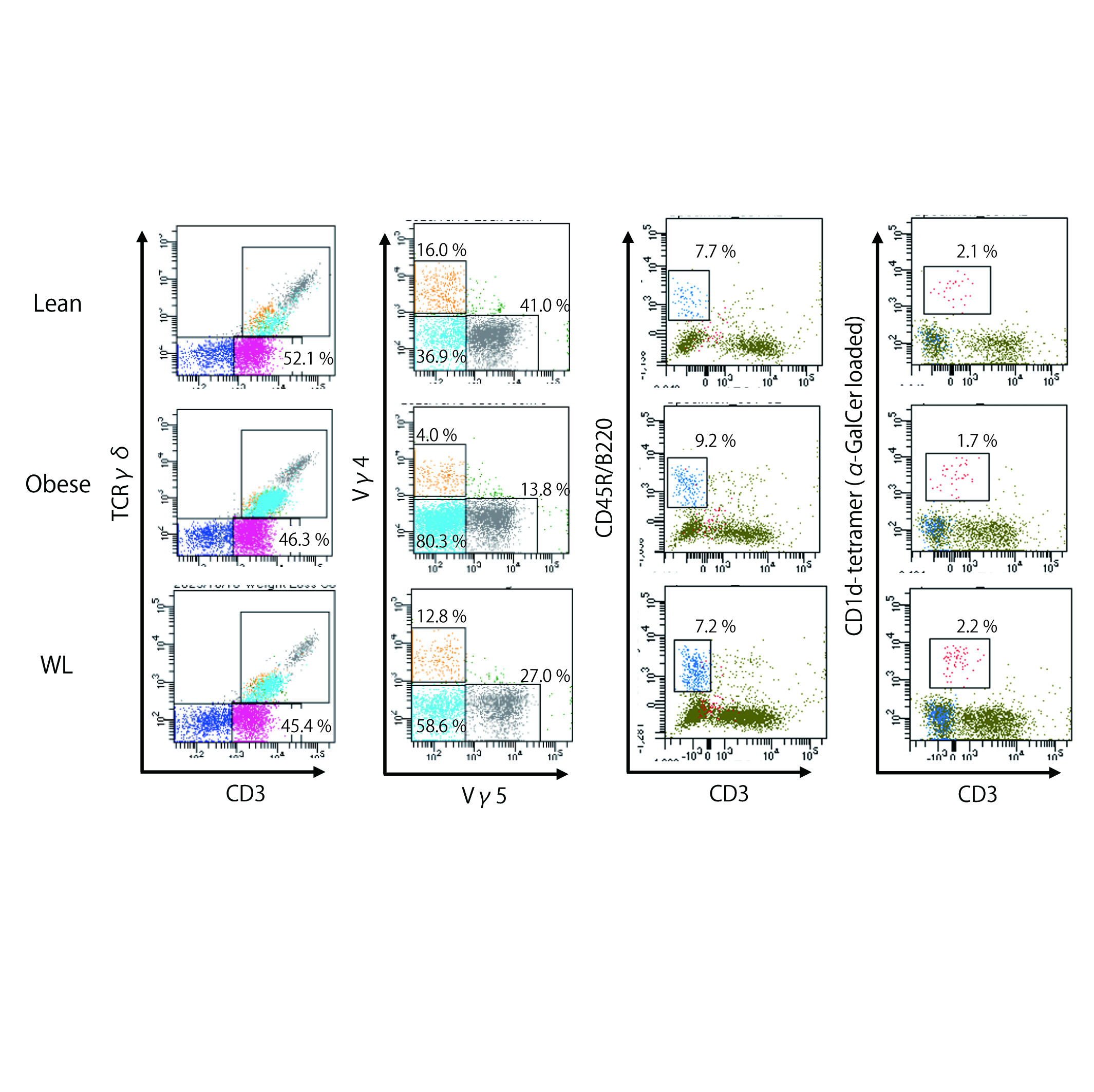

Supplement: Supplementary file 3 — Supporting Information 3 Figure S2: The plots of lymphocyte fraction. The representative plots of T cells, dermal γδ T cells (Vγ4+Vγ5−, Vγ4−Vγ5−), DETCs (Vγ4−Vγ5+), B cells, and iNKT cells in intact skin. [file JIMR-2026-3930910-s003.tif]

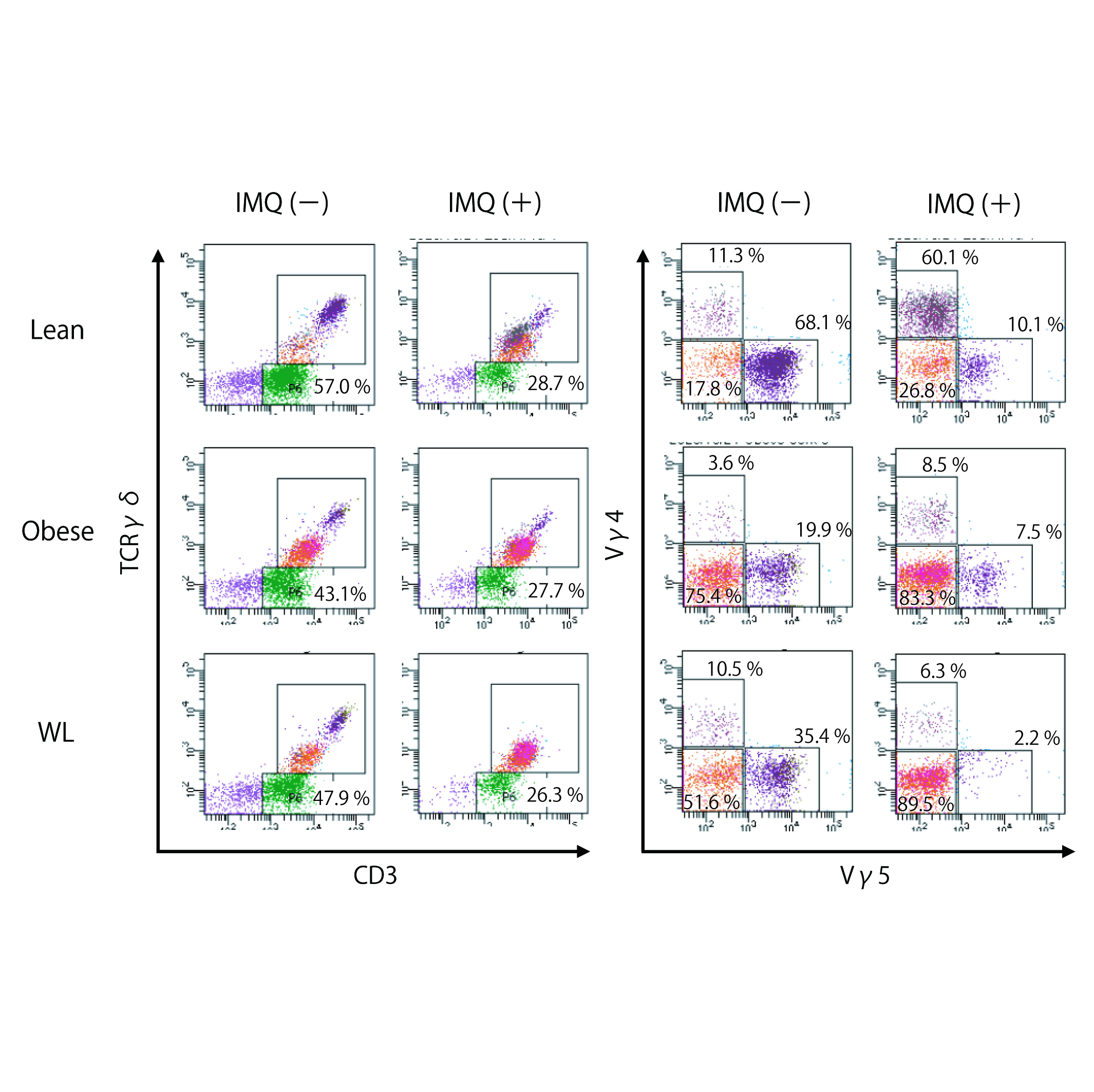

Supplement: Supplementary file 4 — Supporting Information 4 Figure S3: Representative plots of T cell and γδ T cell fractions on day 7 of IMQ application. Representative plots of T cells, dermal γδ T cells (Vγ4+Vγ5−, Vγ4−Vγ5−), and DETCs (Vγ4−Vγ5+) in both IMQ (‐) and IMQ (+) skin. [file JIMR-2026-3930910-s004.tif]
