# Supplementary material for: High Fat Diet‐Induced Obesity Alters Cutaneous Immune Cell Function, and These Changes Persist After Weight Loss
Source: J Immunol Res. 2026 May 15;2026:3930910. doi: 10.1155/jimr/3930910 (PMC13177834; doi:10.1155/jimr/3930910)
Supplement: Supplementary file 2 — Supporting Information 2 Table S1: Detailed results of statistical analyses for all experiments. [file JIMR-2026-3930910-s002.pdf]

Fig.1b Weight (g)

Time Point (week)	Lean Mean ± SD	Obese Mean ± SD	WL Mean ± SD	F value	df (2, 69)	Overall *p*-value	Post-hoc Test (Tukey's HSD) Pairwise Comparisons	Adjusted *p*-value	Conclusion
0	21.1±1.0	21.1±1.1	21.3±1.1	0.174	2, 69	0.8404			
1	22.1±1.1	23.5±1.3	23.4±1.4	8.67	2, 69	0.0004	Lean vs. Obese	0.0008	***
							Lean vs. WL	0.0041	##
							Obese vs. WL	0.8588	N.S.
2	23.0±1.0	24.9±1.6	24.8±1.9	12.08	2, 69	<.0001	Lean vs. Obese	0.0001	***
							Lean vs. WL	0.0003	###
							Obese vs. WL	0.9772	N.S.
3	23.7±1.0	26.2±1.8	26.5±2.3	17.15	2, 69	<.0001	Lean vs. Obese	<.0001	***
							Lean vs. WL	<.0001	###
							Obese vs. WL	0.859	N.S.
4	24.7±1.2	27.6±2.1	28.2±2.6	20.96	2, 69	<.0001	Lean vs. Obese	<.0001	***
							Lean vs. WL	<.0001	###
							Obese vs. WL	0.5695	N.S.
5	25.5±1.3	29.5±2.7	29.9±3.0	23.55	2, 69	<.0001	Lean vs. Obese	<.0001	***
							Lean vs. WL	<.0001	###
							Obese vs. WL	0.8722	N.S.
6	26.0±1.4	30.8±2.9	31.4±3.3	30.44	2, 69	<.0001	Lean vs. Obese	<.0001	***
							Lean vs. WL	<.0001	###
							Obese vs. WL	0.6717	N.S.
7	26.4±1.6	32.2±3.4	32.6±3.4	33.17	2, 69	<.0001	Lean vs. Obese	<.0001	***
							Lean vs. WL	<.0001	###
							Obese vs. WL	0.884	N.S.
8	27.0±1.5	33.5±3.4	34.2±3.5	43.31	2, 69	<.0001	Lean vs. Obese	<.0001	***
							Lean vs. WL	<.0001	###
							Obese vs. WL	0.7018	N.S.
9	27.4±1.7	34.7±3.3	35.6±3.7	53.33	2, 69	<.0001	Lean vs. Obese	<.0001	***
							Lean vs. WL	<.0001	###
							Obese vs. WL	0.5197	N.S.
10	27.6±1.8	36.2±3.9	33.6±4.5	36.53	2, 69	<.0001	Lean vs. Obese	<.0001	***
							Lean vs. WL	<.0001	###
							Obese vs. WL	0.0366	$
11	27.9±1.9	37.0±3.7	31.3±3.2	56.1	2, 69	<.0001	Lean vs. Obese	<.0001	***
							Lean vs. WL	0.0007	###
							Obese vs. WL	<.0001	$$$
12	28.4±1.8	38.4±3.9	30.4±3.9	60.33	2, 69	<.0001	Lean vs. Obese	<.0001	***
							Lean vs. WL	0.1178	N.S.
							Obese vs. WL	<.0001	$$$
13	28.9±1.9	39.8±4.3	30.5±3.8	69.63	2, 69	<.0001	Lean vs. Obese	<.0001	***
							Lean vs. WL	0.2617	N.S.
							Obese vs. WL	<.0001	$$$
14	29.3±2.2	41.0±4.3	30.8±2.8	92.8	2, 69	<.0001	Lean vs. Obese	<.0001	***
							Lean vs. WL	0.2376	N.S.
							Obese vs. WL	<.0001	$$$
15	29.6±2.4	42.4±4.4	30.9±2.7	111.3	2, 69	<.0001	Lean vs. Obese	<.0001	***
							Lean vs. WL	0.382	N.S.
							Obese vs. WL	<.0001	$$$
16	30.0±2.4	43.4±4.5	31.2±2.8	115.14	2, 69	<.0001	Lean vs. Obese	<.0001	***
							Lean vs. WL	0.4809	N.S.
							Obese vs. WL	<.0001	$$$
17	30.4±2.4	44.0±4.2	31.2±2.6	140.9	2, 69	<.0001	Lean vs. Obese	0.663	N.S.
							Lean vs. WL	<.0001	***
							Obese vs. WL	<.0001	$$$
18	30.5±2.6	44.7±4.2	31.3±2.7	145.07	2, 69	<.0001	Lean vs. Obese	<.0001	***
							Lean vs. WL	0.6576	N.S.
							Obese vs. WL	<.0001	$$$

Fig.1b Weight gain ratio (%)

Time Point (week)	Lean Mean ± SD	Obese Mean ± SD	WL Mean ± SD	F value	df (2, 69)	Overall *p* -value	Post-hoc Test (Tukey's HSD) Pairwise Comparisons	Adjusted *p* -value	Conclusion
0	100.0±0.0	100.0±0.0	100.0±0.0	N.S.	2, 69	N.S.	N/A	N/A	N.S.
1	105.0±2.1	111.5±4.0	109.8±3.9	23.62	2, 69	<.0001	Lean vs. Obese	<.0001	***
							Lean vs. WL	<.0001	###
							Obese vs. WL	0.2041	N.S.
2	109.5±2.1	117.9±5.0	116.7±5.7	23.67	2, 69	<.0001	Lean vs. Obese	<.0001	***
							Lean vs. WL	<.0001	###
							Obese vs. WL	0.5916	N.S.
3	113.3±2.7	124.0±5.7	124.3±6.9	32.1	2, 69	<.0001	Lean vs. Obese	<.0001	***
							Lean vs. WL	<.0001	###
							Obese vs. WL	0.9751	N.S.
4	116.8±2.3	130.7±6.5	132.6±8.1	47.52	2, 69	<.0001	Lean vs. Obese	<.0001	***
							Lean vs. WL	<.0001	###
							Obese vs. WL	0.5554	N.S.
5	120.9±2.4	139.6±8.7	140.2±10.2	46.53	2, 69	<.0001	Lean vs. Obese	<.0001	***
							Lean vs. WL	<.0001	###
							Obese vs. WL	0.9549	N.S.
6	122.6±3.6	145.6±9.4	147.6±11.1	61.53	2, 69	<.0001	Lean vs. Obese	<.0001	***
							Lean vs. WL	<.0001	###
							Obese vs. WL	0.7001	N.S.
7	124.0±4.1	152.2±11.3	153.0±11.6	70.56	2, 69	<.0001	Lean vs. Obese	<.0001	***
							Lean vs. WL	<.0001	###
							Obese vs. WL	0.9479	N.S.
8	126.5±4.6	158.5±11.3	160.7±12.5	87.09	2, 69	<.0001	Lean vs. Obese	<.0001	***
							Lean vs. WL	<.0001	###
							Obese vs. WL	0.7272	N.S.
9	128.9±5.5	163.9±10.9	167.3±13.5	98.63	2, 69	<.0001	Lean vs. Obese	<.0001	***
							Lean vs. WL	<.0001	###
							Obese vs. WL	0.4996	N.S.
10	130.6±6.6	171.1±12.5	157.7±17.2	61.74	2, 69	<.0001	Lean vs. Obese	<.0001	***
							Lean vs. WL	0.1118	N.S.
							Obese vs. WL	0.0016	$$
11	131.7±7.1	175.2±13.1	147.2±12.7	91.33	2, 69	<.0001	Lean vs. Obese	<.0001	***
							Lean vs. WL	<.0001	###
							Obese vs. WL	<.0001	$$$
12	133.5±6.6	181.7±12.3	142.7±16.6	100.97	2, 69	<.0001	Lean vs. Obese	<.0001	***
							Lean vs. WL	0.0336	#
							Obese vs. WL	<.0001	$$$
13	135.6±6.7	188.2±13.5	143.3±17.0	112.66	2, 69	<.0001	Lean vs. Obese	<.0001	***
							Lean vs. WL	0.1141	N.S.
							Obese vs. WL	<.0001	$$$
14	137.0±8.0	193.7±13.7	145.2±13.0	160.64	2, 69	<.0001	Lean vs. Obese	<.0001	***
							Lean vs. WL	0.0505	N.S.
							Obese vs. WL	<.0001	$$$
15	139.1±8.9	200.6±14.2	145.4±13.0	191.2	2, 69	<.0001	Lean vs. Obese	<.0001	***
							Lean vs. WL	0.169	N.S.
							Obese vs. WL	<.0001	$$$
16	140.7±9.5	205.3±14.7	146.6±12.6	196.69	2, 69	<.0001	Lean vs. Obese	<.0001	***
							Lean vs. WL	0.2413	N.S.
							Obese vs. WL	<.0001	$$$
17	141.9±9.9	208.3±13.7	146.8±12.3	225.17	2, 69	<.0001	Lean vs. Obese	<.0001	***
							Lean vs. WL	0.3522	N.S.
							Obese vs. WL	<.0001	$$$
18	142.0±10.2	211.6±13.6	147.5±13.4	229.95	2, 69	<.0001	Lean vs. Obese	<.0001	***
							Lean vs. WL	0.2879	N.S.
							Obese vs. WL	<.0001	$$$

Fig.1d ipGTT (blood glucose, mg/dL)

Time Point (min)	Lean Mean ± SD	Obese Mean ± SD	WL Mean ± SD	F value	df (2, 33)	Overall *p* -value	Post-hoc Test (Tukey's HSD) Pairwise Comparisons	Adjusted *p* -value	Conclusion
0	142.2±24.4	201.5±21.3	148.5±20.9	26.6012	2, 33	<.0001	Lean vs. Obese	<.0001	***
							Lean vs. WL	0.9082	N.S.
							Obese vs. WL	<.0001	$$$
15	337.4±87.7	408.0±101.3	317.3±63.5	2.275	2, 33	0.1371	Lean vs. Obese	0.2377	N.S.
							Lean vs. WL	0.9616	N.S.
							Obese vs. WL	0.155	N.S.
30	372.8±75.6	477.8±137.1	402.2±76.9	11.3846	2, 33	0.001	Lean vs. Obese	0.0008	***
							Lean vs. WL	0.3449	N.S.
							Obese vs. WL	0.015	$
60	372.1±161.1	525.0±111.5	424.2±67.3	2.4103	2, 33	0.1237	Lean vs. Obese	0.1081	N.S.
							Lean vs. WL	0.3953	N.S.
							Obese vs. WL	0.6878	N.S.
90	247.5±100.3	477.7±90.2	346.5±72.9	13.1541	2, 33	0.0005	Lean vs. Obese	0.0004	***
							Lean vs. WL	0.0981	N.S.
							Obese vs. WL	0.0288	$
120	228.7±88.2	407.0±69.8	265.7±80.9	17.1733	2, 33	0.0001	Lean vs. Obese	0.0001	***
							Lean vs. WL	0.2139	N.S.
							Obese vs. WL	0.0034	$$

Fig.1f

	Lean Mean ± SD	Obese Mean ± SD	WL Mean ± SD	F value	df (2, 21)	*p* -value	Pairwise Comparisons	*p* -value	Conclusion
Total cholesterol (mg/dL)	115.8±10.9	227.3±19.2	159.4±29.8	55.003	2, 21	<.0001	Lean vs. Obese	<.0001	***
							Lean vs. WL	0.0025	##
							Obese vs. WL	<.0001	$$$
LDL cholesterol (mg/dL)	8.1±1.4	14.9±3.6	10.3±2.3	13.9695	2, 21	0.0001	Lean vs. Obese	<.0001	***
							Lean vs. WL	0.2566	N.S.
							Obese vs. WL	0.0053	$$
HDL cholesterol (mg/dL)	72.1±6.2	86.5±4.3	77.62±8.4	9.8122	2, 21	0.001	Lean vs. Obese	0.0007	***
							Lean vs. WL	0.2362	N.S.
							Obese vs. WL	0.0036	$$

Fig.2

target	Lean Mean ± SD	Obese Mean ± SD	WL Mean ± SD	F value	df (2, 15)	Overall *p* -value	Post-hoc Test (Tukey's HSD) Pairwise Comparisons	Adjusted *p* -value	Conclusion
COL1A1	1.0±0.313	0.47±0.033	0.55±0.197	8.2331	2, 15	0.0049	Lean vs. Obese	0.0093	**
							Lean vs. WL	0.0096	##
							Obese vs. WL	0.8447	N.S.
T-bet	1.0±0.66	0.78±0.424	1.7±1.19	1.683	2, 15	0.2303			
GATA3	1.0±0.13	1.15±0.47	1.23±0.23	0.8545	2, 15	0.4465			
RORγt	1.0±0.27	2.33±0.79	6.83±3.99	8.7557	2, 15	0.0053	Lean vs. Obese	0.024	*
							Lean vs. WL	0.005	##
							Obese vs. WL	0.6039	N.S.
Foxp3	1.0±0.54	1.92±1.05	1.89±0.84	2.3639	2, 15	0.1281			
CD4	1.0±0.53	2.13±1.37	5.14±4.73	2.808	2, 15	0.0999	Lean vs. Obese	0.8096	N.S.
							Lean vs. WL	0.0726	N.S.
							Obese vs. WL	0.257	N.S.
CD8	1.0±0.24	1.11±0.60	4.99±4.30	3.6562	2, 15	0.0606	Lean vs. Obese	0.9978	N.S.
							Lean vs. WL	0.0726	N.S.
							Obese vs. WL	0.1129	N.S.
CCL20	1.0±0.41	4.96±0.42	7.66±5.29	8.2303	2, 15	0.0056	Lean vs. Obese	0.0009	***
							Lean vs. WL	0.0282	#
							Obese vs. WL	0.6908	N.S.
CCR6	1.0±0.05	2.26±1.74	4.56±2.83	3.0055	2, 15	0.095	Lean vs. Obese	0.6195	N.S.
							Lean vs. WL	0.0727	N.S.
							Obese vs. WL	0.2423	N.S.
IFN-γ	Not detected	Not detected	Not detected						
IL-4	1.0±0.31	2.68±2.44	2.35±1.25	1.5622	2, 15	0.2495			
IL-1β	1.0±0.05	1.09±1.06	0.66±0.25	0.3542	2, 15	0.7088			
IL-6	1.0±0.52	0.25±0.14	0.35±0.17	6.0334	2, 15	0.0253	Lean vs. Obese	0.0337	*
							Lean vs. WL	0.0404	#
							Obese vs. WL	0.8796	N.S.
IL-17A	1.0±0.64	2.81±0.79	3.85±2.18	11.5682	2, 15	0.0395	Lean vs. Obese	0.0078	**
							Lean vs. WL	0.0326	#
							Obese vs. WL	0.5121	N.S.
IL-23	Not detected	Not detected	Not detected						
IL-22	1.0±0.73	5.37±5.22	7.41±4.33	4.2294	2, 15	0.0475	Lean vs. Obese	0.1802	N.S.
							Lean vs. WL	0.0392	#
							Obese vs. WL	0.6991	N.S.
TNF-α	1.0±0.71	2.49±1.71	2.78±0.69	3.121	2, 15	0.0844	Lean vs. Obese	0.5624	N.S.
							Lean vs. WL	0.084	N.S.
							Obese vs. WL	0.1101	N.S.
TGF-β	1.0±0.31	1.25±0.38	1.16±0.29	0.7342	2, 15	0.5003			
IL-10	1.0±0.63	0.78±1.74	0.84±0.41	0.2814	2, 15	0.76			
iNOS	1.0±0.49	2.99±1.81	1.99±1.10	4.1519	2, 15	0.0494	Lean vs. Obese	0.0442	*
							Lean vs. WL	0.383	N.S.
							Obese vs. WL	0.439	N.S.
CD11c	1.0±0.29	1.05±0.41	1.61±0.62	2.6112	2, 15	0.118	Lean vs. Obese	0.9835	N.S.
							Lean vs. WL	0.1084	N.S.
							Obese vs. WL	0.211	N.S.
CD206	1.0±0.31	0.57±0.32	0.59±0.11	4.604	2, 15	0.0291	Lean vs. Obese	0.03	*
							Lean vs. WL	0.0367	#
							Obese vs. WL	0.9932	N.S.

Fig.3a

	Lean Mean ± SD	Obese Mean ± SD	WL Mean ± SD	F value	df (2, 15)	Overall p -value	Post-hoc Test (Tukey's HSD) Pairwise Comparisons	Adjusted p -value	Conclusion
Lymphocytes (%)	88.8±1.91	88.6±1.1	89.2±1.7	0.1788	2, 15	0.8381			
T cells (%)	51.2±3.8	44.2±2.6	45.7±6.5	2.8674	2, 15	0.0997			
Vγ4+Vγ5- (%)	16.9±3.3	8.1±2.2	12.6±1.7	12.902	2, 15	0.0023	Lean vs. Obese	0.0018	**
							Lean vs. WL	0.1929	N.S.
							Obese vs. WL	0.0298	$
Vγ4-Vγ5- (%)	27.9±12.5	65.3±11.7	46.6±8.4	10.1726	2, 15	0.0049	Lean vs. Obese	0.004	**
							Lean vs. WL	0.0488	##
							Obese vs. WL	0.2741	N.S.
Vγ4-Vγ5+ (%)	54.5±13.4	25.8±8.1	40.3±7.6	7.8398	2, 15	0.0107	Lean vs. Obese	0.0134	*
							Lean vs. WL	0.8801	N.S.
							Obese vs. WL	0.0281	$
B cells (%)	72.1±6.2	86.5±4.3	77.62±8.4	1.4752	2, 15	0.2601			
iNKT cells (%)	115.8±10.9	227.3±19.2	159.4±29.8	1.1641	2, 15	0.3389			

Fig.3b

	Lean Mean ± SD	Obese Mean ± SD	WL Mean ± SD	F value	df (2, 15)	p -value	Pairwise Comparisons	p -value	Conclusion
Leukocytes ($\times 10^4$)	6.31±2.4	5.7±2.9	5.5±1.8	0.2124	2, 15	0.8112			
Lymphocytes ($\times 10^4$)	5.8±2.1	5.0±2.5	4.8±1.5	0.2069	2, 15	0.8155			
T cells ($\times 10^4$)	3.3±1.4	2.6±1.0	2.3±0.5	1.6839	2, 15	0.2211			
Vγ4+Vγ5- ($\times 10^4$)	0.27±0.08	0.17±0.12	0.20±0.13	1.1096	2, 15	0.357			
Vγ4-Vγ5- ($\times 10^4$)	0.53±0.40	1.96±1.16	1.07±0.19	4.7298	2, 15	0.0358	Lean vs. Obese	0.0292	*
							Lean vs. WL	0.2158	N.S.
							Obese vs. WL	0.4963	N.S.
Vγ4-Vγ5+ ($\times 10^4$)	0.79±0.39	0.59±0.38	0.73±0.41	0.3376	2, 15	0.7191			
B cells ($\times 10^4$)	0.15±0.12	0.14±0.06	0.12±0.09	0.1262	2, 15	0.8823			
iNKT cells ($\times 10^4$)	0.04±0.03	0.02±0.01	0.03±0.02	0.7546	2, 15	0.4872			

Fig.4b PASI socore

Time Point (day)	Lean Mean ± SD	Obese Mean ± SD	WL Mean ± SD	F value	df (2, 15)	Overall *p* -value	Post-hoc Test (Tukey's HSD) Pairwise Comparisons	Adjusted *p* -value	Conclusion
1	0 ± 0	0 ± 0	0 ± 0	N.S	2, 15	N.S.	N/A	N/A	N.S.
2	1.17 ± 1.17	1.67 ± 1.37	1.17 ± 0.75	2.8674	2, 15	0.0997			
3	4.33 ± 2.07	5.83 ± 2.04	4.33 ± 1.03	1.4211	2, 15	0.2722			
4	6.33 ± 1.37	8.00 ± 2.00	8.17 ± 0.98	2.7073	2, 15	0.0991			
5	5.67 ± 1.03	9.50 ± 1.38	11.17 ± 0.75	40.5189	2, 15	<.0001	Lean vs. Obese	<.0001	***
							Lean vs. WL	<.0001	###
							Obese vs. WL	0.0444	$
6	4.17 ± 0.98	8.17 ± 2.93	10.83 ± 1.17	18.5933	2, 15	<.0001	Lean vs. Obese	0.0065	**
							Lean vs. WL	<.0001	###
							Obese vs. WL	0.0692	N.S.
7	4.00 ± 1.41	7.00 ± 2.53	10.17 ± 1.17	17.5256	2, 15	<.0001	Lean vs. Obese	0.0213	*
							Lean vs. WL	<.0001	###
							Obese vs. WL	0.0291	$

Fig.4b TEWL (g/m^2h)

	IMQ (−)			IMQ (+)								
Time Point (day)	Lean Mean ± SD	Obese Mean ± SD	WL Mean ± SD	Lean Mean ± SD	Obese Mean ± SD	WL Mean ± SD	F value	df (5, 30)	Overall *p* -value	Post-hoc Test (Tukey's HSD) Pairwise Comparisons	Adjusted *p* -value	Conclusion
1	26.5 ± 10.9	18.8 ± 1.7	19.5 ± 2.9	22.8 ± 4.0	19.2 ± 4.0	21.2 ± 5.4	1.544	5, 30	0.2072			
2	25.1 ± 9.7	20.1 ± 3.8	21.1 ± 0.6	50.3 ± 10.9	40.7 ± 14.2	55.1 ± 9.6	15.3567	5, 30	<.0001	IMQ (+) Lean vs. Obese	0.5027	N.S.
										IMQ (+) Lean vs. WL	0.9513	N.S.
										IMQ (+) Obese vs. WL	0.1215	N.S.
										IMQ (−) vs. IMQ (+)	0.0137	
3	22.6 ± 4.5	17.3 ± 1.5	22.3 ± 4.9	63.0 ± 3.5	51.7 ± 14.3	74.2 ± 6.8	63.7784	5, 30	<.0001	IMQ (+) Lean vs. Obese	0.1153	N.S.
										IMQ (+) Lean vs. WL	0.1126	N.S.
										IMQ (+) Obese vs. WL	0.0001	$$
										IMQ (−) vs. IMQ (+)	<.0001	
4	20.4 ± 3.5	18.0 ± 3.5	22.0 ± 4.3	71.3 ± 9.6	61.3 ± 11.9	88.1 ± 11.0	79.2773	5, 30	<.0001	IMQ (+) Lean vs. Obese	0.325	N.S.
										IMQ (+) Lean vs. WL	0.0159	#
										IMQ (+) Obese vs. WL	<.0001	$$$
										IMQ (−) vs. IMQ (+)	<.0001	
5	17.8 ± 4.7	15.4 ± 2.2	18.3 ± 3.1	71.3 ± 4.3	59.2 ± 10.0	89.0 ± 6.8	172.9619	5, 30	<.0001	IMQ (+) Lean vs. Obese	0.0157	*
										IMQ (+) Lean vs. WL	0.0002	###
										IMQ (+) Obese vs. WL	<.0001	$$$
										IMQ (−) vs. IMQ (+)	<.0001	
6	17.3 ± 5.0	15.9 ± 4.5	16.9 ± 3.4	65.2 ± 3.7	65.9 ± 10.1	82.0 ± 6.7	146.0183	5, 30	<.0001	IMQ (+) Lean vs. Obese	1	N.S.
										IMQ (+) Lean vs. WL	0.0006	###
										IMQ (+) Obese vs. WL	0.001	$$
										IMQ (−) vs. IMQ (+)	<.0001	
7	16.7 ± 4.1	15.9 ± 2.9	15.7 ± 2.4	69.0 ± 7.4	68.1 ± 10.3	78.3 ± 9.6	113.2379	5, 30	<.0001	IMQ (+) Lean vs. Obese	0.9999	N.S.
										IMQ (+) Lean vs. WL	0.2241	N.S.
										IMQ (+) Obese vs. WL	0.1498	N.S.
										IMQ (−) vs. IMQ (+)	<.0001	

Fig.4c IMQ (－)

	Lean Mean ± SD	Obese Mean ± SD	WL Mean ± SD	F value	df (2, 15)	Overall *p* -value	Post-hoc Test (Tukey's HSD) Pairwise Comparisons	Adjusted *p* -value	Conclusion
T cells (%)	52.9 ± 7.5	40.8 ± 3.7	43.6 ± 0.8	5.0811	2, 15	0.0512	Lean vs. Obese		
							Lean vs. WL		
							Obese vs. WL		
Vγ4+Vγ5- (%)	12.3 ± 2.6	5.6 ± 1.9	11.6 ±2.3	7.8186	2, 15	0.0213	Lean vs. Obese	0.027	*
							Lean vs. WL	0.9394	N.S.
							Obese vs. WL	0.0404	$
Vγ4-Vγ5- (%)	29.2 ± 5.9	66.8 ± 1.4	46.8 ± 3.8	61.9394	2, 15	<.0001	Lean vs. Obese	<.0001	***
							Lean vs. WL	0.0047	##
							Obese vs. WL	0.0025	$$
Vγ4-Vγ5+ (%)	49.1 ± 9.2	18.4 ± 0.4	33.9 ± 3.5	21.8739	2, 15	0.0018	Lean vs. Obese	0.0014	**
							Lean vs. WL	0.0385	#
							Obese vs. WL	0.0363	$

Fig.4c IMQ (＋)

	Lean Mean ± SD	Obese Mean ± SD	WL Mean ± SD	F value	df (2, 15)	Overall *p* -value	Post-hoc Test (Tukey's HSD) Pairwise Comparisons	Adjusted *p* -value	Conclusion
T cells (%)	31.6 ± 6.0	29.2 ± 9.8	35.4 ± 8.7	0.4194	2, 15	0.6754			
Vγ4+Vγ5- (%)	58.1 ± 3.7	11.1 ± 3.7	11.3 ± 4.1	150.4443	2, 15	<.0001	Lean vs. Obese	<.0001	***
							Lean vs. WL	<.0001	###
							Obese vs. WL	0.9969	N.S.
Vγ4-Vγ5- (%)	28.4 ± 3.3	78.8 ± 1.9	80.7 ± 5.2	191.708	2, 15	<.0001	Lean vs. Obese	<.0001	***
							Lean vs. WL	<.0001	###
							Obese vs. WL	0.8052	N.S.
Vγ4-Vγ5+ (%)	7.1±3.2	5.4±2.3	2.0±0.8	3.7867	2, 15	0.0864			

Fig.4d IMQ (－)

	Lean Mean ± SD	Obese Mean ± SD	WL Mean ± SD	F value	df (2, 15)	Overall *p* -value	Post-hoc Test (Tukey's HSD) Pairwise Comparisons	Adjusted *p* -value	Conclusion
T cells ($\times 10^4$)	2.3± 0.6	1.8± 0.2	2.1± 0,4	1.0775	2, 15	0.3983			
Vγ4+Vγ5- ($\times 10^4$)	0.16± 0.04	0.10± 0.04	0.20± 0.05	3.5587	2, 15	0.0957			
Vγ4-Vγ5- ($\times 10^4$)	0.41± 0.18	1.25± 0.31	0.80± 0.01	12.9033	2, 15	0.0067	Lean vs. Obese	0.0055	**
							Lean vs. WL	0.0985	N.S.
							Obese vs. WL	0.119	N.S.
Vγ4-Vγ5+ ($\times 10^4$)	0.71± 0.35	0.35± 0.09	0.59± 0.09	2.2285	2, 15	0.1889			

Fig.4d IMQ (＋)

	Lean Mean ± SD	Obese Mean ± SD	WL Mean ± SD	F value	df (2, 15)	Overall *p* -value	Post-hoc Test (Tukey's HSD) Pairwise Comparisons	Adjusted *p* -value	Conclusion
T cells ($\times 10^4$)	1.4± 0.32	1.1± 0.5	1.4± 0.14	0.5298	2, 15	0.6139			
Vγ4+Vγ5- ($\times 10^4$)	1.52± 0.81	0.21± 0.07	0.19± 0.04	7.8242	2, 15	0.0213	Lean vs. Obese	0.0335	*
							Lean vs. WL	0.0314	#
							Obese vs. WL	0.9983	N.S.
Vγ4-Vγ5- ($\times 10^4$)	0.72± 0.29	1.51± 0.29	1.45± 0.45	4.6663	2, 15	0.0599	Lean vs. Obese	0.0747	N.S.
							Lean vs. WL	0.0939	N.S.
							Obese vs. WL	0.975	N.S.
Vγ4-Vγ5+ ($\times 10^4$)	0.20± 0.16	0.10± 0.05	0.03± 0.01	2.3216	2, 15	0.1792			
